# Takeaway food consumption and depressive symptoms in Chinese university students: mediating effects of physical activity

**DOI:** 10.3389/fpsyt.2024.1450718

**Published:** 2025-01-16

**Authors:** Jianyu Tan, Rui Wang, Zhewei Su, Yiting Kong, Pan Ran, Andrew Greenshaw, Su Hong, Qi Zhang, Wo Wang, Ming Ai, Li Kuang

**Affiliations:** ^1^ Department of Psychiatry, The First Affiliated Hospital of Chongqing Medical University, Chongqing, China; ^2^ Psychiatric Center, The First Affiliated Hospital of Chongqing Medical University, Chongqing, China; ^3^ Department of Psychiatry, University of Alberta, Edmonton, AB, Canada; ^4^ Mental Health Center, University-Town Hospital of Chongqing Medical University, Chongqing, China

**Keywords:** takeaway, mediation effect, depressive symptoms, physical activity, college students

## Abstract

**Background:**

The consumption of takeaways is becoming increasingly prevalent. Despite this, the relationship between takeaway food consumption and depressive symptoms in Chinese populations has not been clarified. Furthermore, the factors that mediate the association between takeaway frequency and depressive symptoms are unknown.

**Methods:**

Questionnaires were employed to collect data from 6,417 new students at Chongqing Medical University in the autumn of 2023, including sociodemographic information, takeaway frequency, physical activity levels (measured by the International Physical Activity Questionnaire Short Form), and depressive symptoms (measured by the Patient Health Questionnaire-9). Multiple linear regression and mediation analysis were performed. Multiple imputations were used to fill in missing data through sensitivity analyses.

**Results:**

Among 6417 participants, 2,606 (40.6%) students ordered takeaway at least once a week, with 235 (3.7%) of them ordering takeaway food every day. Takeaway frequency was significantly associated with depressive symptoms (β=0.034, P=0.006), and physical activity partially mediated this relationship (95% bootstrap confidence interval=0.0024, 0.0371).

**Conclusions:**

The study highlights the negative relationship between takeaway frequency and emotional well-being, emphasizing the need to focus on the emotional health of frequent takeaway food consumers. Moreover, our study suggests that increased physical activity may alleviate takeaway-induced mood-related outcomes.

## 1 Introduction

The takeaway industry has witnessed significant growth in recent years owing to the rapid development of the social economy and online business (1). The popularity of takeaway food is due to its convenience and time-saving advantage (2, 3). Moreover, the Covid-19 pandemic further triggered in the creation of several takeaway platforms, driven by a substantial urban customer base (4). In 2022, the number of users on all takeaway platforms in China was about 520 million (5). Among the consumers who ordered takeaway during the same year, 50.38% indulged in takeaway at least once daily (6).

Chinese college students commonly reside in university dormitories (7). Despite the availability of dining facilities provided by universities, the variety of takeaway food and the convenience of takeaway food ordering has prompted a significant number of students to order takeaway food (8). A survey of Chinese young people revealed that 15.4% order takeaway at least once a week (9). This dietary pattern has often been regarded as unhealthy due to its high energy, fat, salt, and sugar content and low vitamin and mineral intake (10, 11). Moreover, the convenience of takeaway food being delivered directly to dormitory floors reduces the likelihood of students dining out. Research indicated a direct correlation between takeaway food consumption and sedentary behavior (8). It is therefore necessary to examine the potential health implications of consuming takeaway food.

However, few studies have explored the emotional health implications of takeaway transactions on customers. A Chinese college students survey conducted in 2018 found that those who ordered takeaway more frequently had higher depression scores (12), but the detailed results and methodology are not accessible. A cohort study conducted in Australia showed that Western dietary patterns at the age of 14, which included high consumption of red meat, takeaways, refined foods, and sweets, were linked to more severe depressive symptoms at the age of 17 (13). However, this study did not specifically focus on takeaway behaviors but rather considered them as part of Western dietary patterns. This highlights the need to explore the impact of takeaway habits on mood, especially among Chinese younger people (12).

Previous research indicated that takeaway consumption may reduce physical activity. An Australian study showed that individuals who ordered takeaway twice a week or more tend to spend more time sitting than those who order only once a week (14). Moreover, individuals who order takeaways may have a lower level of physical activity and tend to spend more time sitting compared to those who cook their meals or dine out.

A correlation between physical activity and depressed mood has been documented in several studies. The result of a meta-analysis, which included 15 longitudinal studies, indicated that compared to adults who did not engage in any physical activity, those who completed half of the recommended amount of physical activity per week (4.4 marginal metabolic equivalent task hours per week [mMET-h/wk]) had an 18% lower risk of depression. Moreover, adults who reached 8.8 mMET-h/wk experienced a 25% reduction in the risk of depression (15). Based on findings from prior research, we speculate that frequent consumption of takeaway meals might elevate the risk of depression, with physical activity potentially exerting a significant influence on this correlation. To the best of our knowledge, no study has investigated the relationship between the frequency of takeaway food consumption and the presence of depressive symptoms. Furthermore, no research has employed mediation analysis to examine the mediating of physical activity on the association between these two factors. Therefore, this study aimed to: (1) explore the relationship between takeaway frequency and depressive symptoms and (2), investigate the mediating role of physical activity in the relationship between takeaway frequency and depressive symptoms.

## 2 Methods

### 2.1 Data collection and subjects

In this cross-sectional study, freshmen of Chongqing Medical University were recruited in 2023 Fall. All participants provided written informed consent prior to their inclusion in the study. They were subsequently requested to complete a questionnaire. A total of 6,417 students completed the valid scale, with 260 students excluded due to illness, absence from school, refusal to complete, etc. The study was approved by the Ethics Committee of the National Clinical Medical Research Center, Second Xiangya Hospital, Central South University (NO.2022S010).

### 2.2 Measures

#### 2.2.1 Sociodemographic characteristics

The following sociodemographic characteristics were collected as control variables: age, gender (male or female), ethnicity (Han or other national minority), only child status (only child or non-only child), the condition of residence (rural or urban), religion(with or without), parental marriage status(single parent family or non-single parent family), family history of mental illness(with or without), physical disease(with or without any diabetes, cardiovascular, liver, kidney, or other physical disease), current smoking(yes or no), and current drinking(yes or no).

#### 2.2.2 Depressive symptoms

Depressive symptoms were analyzed using the Chinese version of the Patient Health Questionnaire for depression (PHQ-9). The scale consists of nine items, with each item having four answer choices: “none at all,” “occasional days,” “more than half of the days,” and “almost every day.” Each choice corresponds to a score of 0, 1, 2, or 3, respectively. The total score ranges from 0 to 27 points, with higher scores indicating a greater likelihood of depression. In China, the scale has been widely employed, demonstrating both high reliability and validity (16, 17). The Cronbach’s alpha coefficient of the scale is 0.84 (18).

#### 2.2.3 Takeaway frequency

The frequency of takeaway orders was assessed using a single question: “Do you usually order takeaway food for your meals?” The answer included the following five choices; “never,” “1-3 times/month,” “1-2 times/week,” “3-5 times/week,” “almost every day”.

#### 2.2.4 Physical activity

Physical activity levels were explored using the Chinese version of the International Physical Activity Questionnaire Short Form (IPAQ-SF) (19, 20). The IPAQ-SF assesses physical activity levels based on seven items that evaluate four distinct types of activity conducted during the past week: vigorous-intensity activity, moderate-intensity activity, light-intensity activity, and sitting behavior. Physical activity was determined based on metabolic equivalents (MET). Previous studies have defined 1 MET as the amount of oxygen consumed at rest. Vigorous-intensity activity corresponds to 8 METs, whereas moderate-intensity activity is equivalent to 4 METs, and light-intensity activity corresponds to 3.3 METs (21). The total physical activity level was calculated by multiplying the MET assignment corresponding to a physical activity of a certain intensity by the weekly frequency of that physical activity and the time spent exercising per day following the scoring protocol (22). The reliability and validity of the Chinese version of the IPAQ-SF employed in this study have been confirmed in various studies (22–24). In this study, 2582 students completed the IPAQ-SF questionnaire. Compared to non-respondents, respondents were older, less likely to be female, had lower depression scores, and there was no difference in the frequency of takeaway ordering.

#### 2.2.5 Statistical analysis

Data analysis was conducted using SPSS 27.0, including the PROCESS macro for mediation analysis. Categorical variables were presented using numerical values and percentages. Given that the PHQ-9 scores were not normally distributed, the data were expressed as medians (quartiles) and the PHQ-9 scores were compared between groups using the Mann-Whitney U test. To explore the relationship between takeaway frequency and depressive symptoms, we employed linear regression analysis.

Utilizing the PROCESS macro, mediation analyses were conducted to analyze whether the relationship between takeaway frequency and depressive symptoms was mediated by physical activity. To best test the mediation effect, the bootstrap method was used to estimate the indirect effect and calculate the 95% confidence interval (CI). The number of bootstrap samples was 5000. Previous literature indicates that a mediation effect is considered significant when the confidence interval (CI) does not include zero for both the lower limit (LLCI) and upper limit (ULCI) (25, 26). The mediation model included sociodemographic characteristics as covariates.

In the sensitivity analysis, missing values were imputed using multiple imputations to test the influence of the missing data. This method generates five imputed datasets, where missing values are replaced with plausible estimates based on the observed data. Multiple linear regression adjusted for model 3 and mediation analysis were then performed on the filled dataset to verify the robustness of the results. Two-tailed p-value <0.05 was considered statistically significant.

## 3 Results

### 3.1 Demographic characteristics of participants

This survey enrolled 6,417 students, with a median age of 19 years (interquartile range: 18-23). Among them, there were 2345 (36.5%) males and 4072 (63.5%) females. **Table 1** displays the demographic characteristics of the participants and the disparities in PHQ-9 scores between various groups. According to sociodemographic classification, individuals who are female, younger, non-only children, reside in rural areas, adhere to a religion, come from single-parent households, have a family history of mental illness, suffer from physical ailments, and are current drinkers were more likely to experience severe depressive symptoms.

**Table 1:** Sociodemographic characteristics and differences in PHQ-9 scores between groups.

Variables	n (%)	PHQ-9scores	P
Age			<0.001
15-17	231 (3.8%)	6 (3,8)	
18-24	5321 (82.9%)	5 (2,7)	
25-	865 (13.3)	3 (0,6)	
Gender			<0.001
male	2345 (36.5%)	4 (1,7)	
female	4072 (63.5%)	5 (2,7)	
Ethnicity			0.055
Han	5824 (90.8%)	4 (2,7)	
other national minority	556 (8.7%)	5 (2,7)	
Only child status			<0.001
only child	2504 (39%)	4 (1,7)	
non-only child	3900 (60.8%)	5 (2,7)	
Residence			<0.001
rural	1277 (20%)	5 (2,7)	
urban	5121 (80%)	4 (2,7)	
Religion			<0.001
with	199 (3.1%)	6 (3,8)	
without	6218 (3.1%)	4 (2,7)	
Parental marriage status			0.004
single parent family	418 (6.5%)	5 (2,8)	
non-single parent family	5999 (93.5%)	4 (2,7)	
Family history of mental illness			<0.001
with	240 (3.7%)	6 (3,8)	
without	6170 (96.2)	4 (2,7)	
Physical disease			<0.001
with	230 (3.6%)	6 (3,9)	
without	6185 (96.4%)	4 (2,7)	
Current smoking			0.806
yes	285 (4.4%)	4 (1,7)	
no	6132 (95.6%)	5 (2,7)	
Current drinking			<0.001
yes	3648 (56.8%)	5 (2,7)	
no	2769 (43.2%)	4 (2,7)	
Takeaway frequency			0.02
never	736 (11.5%)	4 (1,7)	
1-3 times per month	3060 (47.7%)	5 (2,7)	
1-2 times per week	1772 (27.6%)	4 (2,7)	
3-5 times per week	599 (9.3%)	4 (2,7)	
everyday	235 (3.7%)	5 (3,7)	

The Mann-Whitney U test was used to explore the differences in PHQ-9 scores between the groups; PHQ-9 scores ≥5 indicate mild and above depressive symptoms.

### 3.2 Relationship between takeaway frequency and depressive symptoms

Among the respondents, 2,606 (40.6%) order takeaway at least once a week, with 235 (3.7%) of them ordering takeaway food every day. Through stepwise multiple linear regression analysis, we investigated the correlation between takeaway frequency and depressive symptoms. In Model 1, we considered takeaway frequency as the independent variable and depressive symptoms as the dependent variable. The analysis revealed a significant association between takeaway frequency and depressive symptoms (B=0.135, β=0.034, P=0.006). In Model 2, sociodemographic characteristics (age, gender, only child status, residence, religion, parent’s marital, family history of mental illness, physical disease, and drinking status) were selected as the independent variables (based on the one-way analyses, two factors were excluded: ethnicity and current smoking status). The relationship between takeaway frequency and depressive symptoms remained significant (β=0.075, *P*<0.001). Moreover, depressive symptoms were significantly correlated with age, gender, only child status, condition of residence, religion, parental marriage status, family history of mental illness, history of physical disease, smoking, and drinking status. In Model 3, physical activity level (expressed as MET) was included. The relationship between takeaway frequency and depressive symptoms remained significant (β=0.084, *P*<0.001, **Table 2**).

**Table 2:** Associations between takeaway frequency, depressive symptoms, and other factors.

Variables	Model 2(F=36.589, adj R^2^ = 0.073)			Model 3(F=11.872, adj R^2^ = 0.060)		
	B	Standardized Coefficient (β)	P	B	Standardized Coefficient (β)	P
Takeaway frequency	0.291	0.075	<0.001	0.333	0.084	<0.001
Age	-0.235	-0.216	<0.001	-0.188	-0.178	<0.001
Gender(female=1)	0.750	0.096	<0.001	0.614	0.078	<0.001
Only child status (non-only child=1)	0.208	0.027	0.038	0.166	0.022	0.297
Residence(rural=1)	0.416	0.046	<0.001	0.13	0.014	0.509
Religion(religious=1)	0.813	0.038	0.003	0.849	0.037	0.064
Parental’s marital (non-single parent=1)	-0.402	-0.027	0.032	-0.304	-0.02	0.33
Family history of mental illness(without=1)	-1.205	-0.062	<0.001	-1.604	-0.085	<0.001
Physical disease(without=1)	-1.655	-0.082	<0.001	-1.155	-0.056	0.005
Current alcohol drinking(no=1)	-0.821	-0.111	<0.001	-1.002	-0.133	<0.001
Physical activity (MET-min/w)				-0.0001	-0.049	0.017

In addition to takeaway frequency, Model 2 adjusted for age, gender, only child status, residence, religion, parent’s marital, family history of mental illness, physical disease, drinking status.

Model3 further adjusted physical activity level.

### 3.3 Mediating role of physical activity level in the relationship between takeaway frequency and depressive symptoms

**Figure 1:**
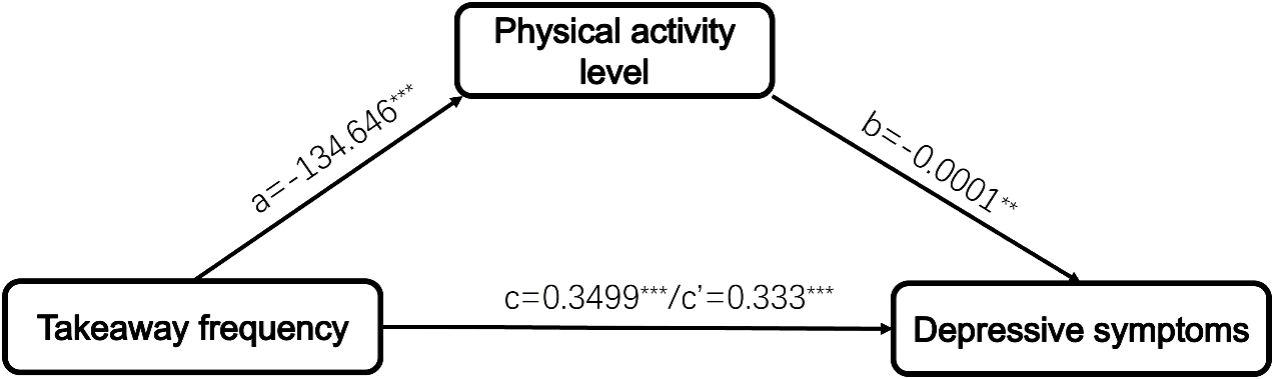
The framework for physical activity level mediating the relationship between takeaway frequency and depressive symptoms. ^∗∗∗^P < 0.001, ^∗∗^P < 0.01.

To examine the role of physical activity as a mediator in the relationship between takeaway frequency and depressive symptoms, we utilized a bootstrapping procedure. The mediation analysis results are presented in **Table 3** after controlling for sociodemographic characteristics (age, gender, ethnicity, only child status, condition of residence, religion, parental marriage status, family history of mental illness, history of physical disease, smoking, and drinking status). A significant negative correlation was observed between takeaway frequency and the physical activity pathway (β=-0.0867, P<0.0001). The total effect of takeaway frequency on depressive symptoms was statistically significant (β=0.0787, P<0.0001). After including both takeaway frequency and physical activity in the model, a significant association was obtained between both takeaway frequency (β=0.084, P=0.0001) and physical activity (β=-0.049, P=0.017) with depressive symptoms. The bootstrap procedure results (**Table 4**) suggested that the association between takeaway frequency and depressive symptoms was partially mediated by physical activity level (indirect effect=0.0169, *SE*=0.0089, 95%CI [0.0024, 0.0371]). The mediating effect contributes to 4.83% of the total effect. To elucidate the relationship between takeaway frequency, physical activity level, and depressive symptoms further, we constructed a mediating effect model (**Figure 1**).

**Table 3:** Results of the Mediation Analysis.

	B	SE	β	t	P
Takeaway frequency→Physical activity					
Takeaway frequency	-134.646	32.227	-0.0867	-4.178	<0.0001
Takeaway frequency→Depressive symptoms					
Takeaway frequency	0.350	0.083	0.0787	4.241	<0.0001
Takeaway frequency, Physical activity→Depressive symptoms					
Takeaway frequency	0.333	0.083	0.084	4.026	0.0001
Physical activity	-0.0001	0.0001	-0.049	-2.38	0.017

SE, Standard Error.

**Table 4:** Mediating model examination by bootstrap.

Path	β	SE	95%CI	
			Lower	Upper
Total effect	0.3499	0.0825	0.1881	0.5117
Direct effect	0.3330	0.0827	0.1708	0.4952
Indirect effect	0.0169	0.0089	0.0024	0.0371

SE, Standard Error.

### 3.4 Sensitivity analysis

In sensitivity analysis, we performed multiple linear regression adjusted for Model 3. The results (in **Supplementary Table 1**) were consistent with the main results that takeaway frequency was also significantly associated with depressive symptoms (B=0.253, P<0.001). The mediation analysis results (**Supplementary Table 2**) were also consistent with previous finding (indirect effect=0.0105, *SE*=0.0041, 95%CI [0.0032, 0.0191]).

## 4 Discussion

In this cross-sectional study, we found that takeaway consumption frequency was significantly positively correlated with depressive symptoms after accounting for multiple sociodemographic characteristics. The relationship between takeaway frequency and depressive symptoms was significantly mediated by physical activity level.

The strong relationship between takeaway consumption frequency and depressive symptoms was also observed in a large survey involving Chinese college students. The survey, which was reported as news rather than a study, showed that approximately 43.2% of college students ordered takeout at least once a week, with around 6.4% ordering takeout nearly every day. Those with more frequent takeaway orders also exhibited higher depression scores (12). A limitation of this study is that its findings were published as news, with no access to detailed results or methodology, limiting its reliability assessment. However, it suggests a link between takeaway consumption and depressive symptoms, highlighting the need for further investigation in college students. Shakya et al. analyzed 1743 participants aged ≥ 24 years and also reported a significant association between takeaway food consumption and depressive symptoms (27).

The adverse effects of takeaway food consumption on mood may be attributed to a multitude of underlying mechanisms. The first possible reason is the nutritional and health issues of the takeaway food itself. In a recent study conducted across 10 major cities in China, researchers investigated the nutritional quality of takeaway food. They found that majority of the top 10 best-selling takeaway dishes have low nutrient quality (28). A study conducted in New Zealand analyzed takeaway food menus from popular online takeaway platforms and found that 90% of the options were unhealthy. These foods were rich in saturated fats, sugars, sodium, and/or alcohol, and deficient in dietary fiber (29). In 2018, a longitudinal study suggested that takeaway behaviors contributed to obesity and inflammation, which in turn caused depressive symptoms (13). The nutritional composition of the diet, encompassing vitamins, fiber, micronutrients, and unsaturated fatty acids, greatly influences emotional well-being (30, 31).

Secondly, takeaway food containers are often made of petroleum-based plastics. Evidence from prior studies indicated that microplastics can be released from carrier container into food, and pose a potential health threat due to polymer degradation (1, 32). In a study conducted by Zhou et al., it was observed that individuals who frequently order takeaway food (approximately 5-10 times per month) may inadvertently consume a range of 145 to 5520 microplastic particles originating from food containers (33). Microplastics, tiny particles originating from various sources, will accumulate in body organs, tissues, and cells. Their presence aggravates the toxicological and pathological reactions within organisms (34–36). Consequently, oxidative stress (37, 38), inflammation (39), and metabolic disturbances (40, 41) may intensify, damaging the emotional well-being (42–44).

Thirdly, frequent takeaway consumption may limit real-life social interactions. Individuals who regularly order takeaway food often exhibit tendencies toward staying indoors, self-isolation, and a reluctance to engage with others, thereby increasing the risk of depression (45–47).

Given the multifaceted nature of takeaway habits, identifying the exact mechanism linking takeaway to depression is difficult. This aspect may only be comprehensively investigated in future prospective studies that meticulously control for confounding factors. In this study, we tested the mediating role of physical activity on the relationship between the takeaway frequency and depressive symptoms. Results indicated that physical activity partially explains the relationship between them. In the mediation analysis, there was a significant negative association between takeaway frequency and physical activity (β=-0.0867, P<0.0001). Previous studies have documented a similar relationship. A study conducted in China analyzed the physical activity habits and takeaway frequency of 2,130 college students. It was found that students who had higher take-out consumption tended to be less physically active and more sedentary (8). In another study, people who order takeaway twice a week tend to spend more time sitting than those who order less frequently (48). Many previous studies have reported a negative relationship between physical activity and symptoms of depression (15, 49). Although the mediating effect of physical activity accounted for only 4.83% of the total effect, it highlights a potential mechanism through which takeaway frequency may influence depressive symptoms. This suggests that promoting physical activity could help mitigate the mental health impacts of frequent takeaway consumption. However, further research is needed to explore other potential mechanisms, such as dietary patterns, sleep quality, and social isolation.

This study has the following strengths. To our knowledge, this is the first study to investigate the relationship between takeaway frequency and depressive symptoms among Chinese college students, while considering several confounding factors. In addition, this is also the first study to explore the mediating role of physical activity on the relationship between takeaway frequency and depressive symptoms.

There were several limitations in this study. Firstly, the use of data from a cross-sectional study makes it impossible to establish causality. Secondly, the study was conducted at a single center, suggesting that the findings may not be universally applicable. Thirdly, a significant number of participants were excluded due to invalid IPAQ questionnaire responses. The excluded population was found to have higher PHQ-9 scores, potentially biasing the outcomes. In a sensitivity analysis, we repeated the main analysis after multiple imputations of missing values, yielding similar results and demonstrating the robustness of the findings. Fourthly, the study only captured data on the frequency of takeaway consumption, without collecting information on the reasons for ordering takeaways or the specific food choices made. This limitation prevented a comprehensive examination of the relationship between various aspects of takeaway behavior and mental health. Future research designs in this area could consider the collection of additional data for analysis.

In conclusion, a significant association was found between takeaway frequency and depressive symptoms. Physical activity partially mediated this relationship. Our results highlight the potential negative effect of takeaway consumption on emotional well-being and recommends that stakeholders should monitor the emotional health of frequent takeaway consumers. In addition, the results indicate that increasing physical activity may alleviate the negative impact of frequent takeaway consumption on mood.

## Data Availability

The datasets used and analyzed during the current study are available from the corresponding author on reasonable request.
