# Supplementary material for: Takeaway food consumption and depressive symptoms in Chinese university students: mediating effects of physical activity
Source: Front Psychiatry. 2025 Jan 16;15:1450718. doi: 10.3389/fpsyt.2024.1450718 (PMC11780376; doi:10.3389/fpsyt.2024.1450718)
Supplement: Supplementary file 1 [file Table1.docx]

**Supplementary table 1.** Associations between takeaway frequency, depressive symptoms, and other factors after multiple imputation

| Variables | B | P |  |
| --- | --- | --- | --- |
|  |  |  |  |
| takeaway frequency | 0.253 | *<0.001* |  |
| age | -0.241 | *<0.001* |  |
| gender(female=1) | 0.769 | *<0.001* |  |
| ethnicity(minority=1) | 0.151 | *<0.001* |  |
| only-child status (not only child=1) | 0.187 | 0.053 |  |
| residence(rural=1) | 0.452 | *<0.001* |  |
| religion(religious=1) | 0.788 | 0.002 |  |
| parent's marital(married=1) | -0.47 | 0.01 |  |
| family history of mental illness(without=1) | -1.011 | *<0.001* |  |
| physical disease(without=1) | -1.706 | *<0.001* |  |
| current smoking(no=1) | -0.421 | 0.061 |  |
| current alcohol drinking(no=1) | -0.826 | *<0.001* |  |
| BMI(kg/m²) | 0.003 | 0.875 |  |
| physical activity (MET-min/w) | -0.0001 | 0.013 |  |

Note: Linear regression analysis was used to analyze.

**Supplementary table 2.** Results of Bootstrap after multiple imputation

| Path | β | SE | 95%CI | |
| --- | --- | --- | --- | --- |
|  |  |  | Lower | Upper |
| Total effect | 0.2675 | 0.0498 | 0.1699 | 0.3652 |
| Direct effect | 0.257 | 0.0499 | 0.1592 | 0.3528 |
| Indirect effect | 0.0105 | 0.0041 | 0.0032 | 0.0191 |

Note: SE = Standard Error.
